# Identification of ethyl pyruvate as a NLRP3 inflammasome inhibitor that preserves mitochondrial integrity

**DOI:** 10.1186/s10020-018-0006-9

**Published:** 2018-03-15

**Authors:** Sujun Li, Fang Liang, Kevin Kwan, Yiting Tang, Xiangyu Wang, Youzhou Tang, Jianhua Li, Huan Yang, Sangeeta S. Chavan, Haichao Wang, Ulf Andersson, Ben Lu, Kevin J. Tracey

**Affiliations:** 1 Department of Hematology and Key Laboratory of non-resolving inflammation and cancer of Human Province, The 3rd Xiangya Hospital, Central South University, Changsha, Hunan province 410000 People’s Republic of China; 2 Laboratory of Biomedical Science, Feinstein Institute for Medical Research, 350 Community Drive, Manhasset, NY 11030 USA; 3 Department of Physiology, School of Basic medical research, Central South University, Changsha, Hunan province People’s Republic of China; 4 Key Laboratory of Medical Genetics, School of Biological Science and Technology, Central South University, Changsha, Hunan province 410000 People’s Republic of China; 5 Department of Emergency Medicine, North Shore University Hospital, Manhasset, NY 11030 USA; 6 Department of Women’s and Children’s Health, Karolinska Institute, 171 76 Stockholm, Sweden

**Keywords:** Ethyl pyruvate, The NLRP3 inflammasomes, Mitochondrial damage, HMGB1, Interleukin-1beta

## Abstract

**Background:**

The NLRP3 inflammasome, a cytosolic complex that mediates the maturation of IL-1β and IL-18 as well as the release of high mobility group box 1 (HMGB1), contributes to the lethality of endotoxic shock. Ethyl pyruvate (EP) was previously shown to inhibit HMGB1 release and promote survival during endotoxemia and experimental sepsis. However, the underlying protective mechanism remains elusive.

**Result:**

EP dose-dependently inhibited the ATP-, nigericin-, alum-, and silica-induced caspase-1 activation and HMGB1 release in mouse macrophages. EP failed to inhibit DNA transfection- or Salmonella Typhimurium-induced caspase-1 activation and HMGB1 release. Mechanistically, EP significantly attenuated mitochondrial damage and cytoplasmic translocation of mitochondrial DNA, a known NLRP3 ligand, without influencing the potassium efflux, the lysosomal rupture or the production of mitochondrial reactive oxygen species (mtROS).

**Conclusion:**

Ethyl pyruvate acts as a novel NLRP3 inflammasome inhibitor that preserves the integrity of mitochondria during inflammation.

## Background

Macrophages, the cells at the front line of defense against infection, express pattern-recognition receptors (PRRs) to detect various inflammatory motifs. PRRs include the membrane-bound Toll-like receptors (TLRs) and NOD-like receptors (NLRs), which scan foreign invaders or sterile tissue damages for pathogen-associated (PAMPs) or damage-associated (DAMPs) molecular patterns. Many NLR family member have been reported to exhibit inflammasome activity in vitro. For instance, NLR family, pyrin domain containing 3 (NLRP3) acts as danger sentinel that self-oligomerize via homotypic NACHT domain interactions to form high-molecular weight complexes that trigger caspase-1 autoactivation (Schroder & Tschopp, 2010).

The NLRP3 inflammasome, an intracellular protein complex consisting of NLRP3, ASC and Caspase-1, controls the activation of caspase-1 and the maturation of the pro-inflammatory cytokines interleukin (IL)-1β and IL-18 (Mariathasan et al., 2006). Accumulated evidence show that the NLRP3 inflammasome also mediates the release of high mobility group box 1 (HMGB1), a late mediator of lethal sepsis, and contributes to the pathogenesis of septic shock (Lu et al., 2012; Lamkanfi et al., 2010; Qin et al., 2006; Rittirsch et al., 2008; Wang et al., 2004; Wang et al., 1999). HMGB1 is an evolutionarily conserved and abundantly expressed nuclear and cytoplasmic protein. Under the physiological condition, HMGB1 predominantly locates in the nucleus due to its two nuclear location sequences (NLSs) in most cell types (Lu et al., 2012; Andersson & Tracey, 2011). Infectious agents or molecules released from damaged cells, such as lipopolysaccharide (LPS), a major cell wall component of gram negative bacteria, ATP or monosodium uric acid crystal (MSU) could induce the translocation of HMGB1 from the nucleus to the cytoplasm and the subsequent release of HMGB1 (Lu et al., 2012; Andersson & Tracey, 2011). Extracellular HMGB1 exerts a variety of biological function by engaging multiple receptors. Though the released HMGB1 might facilitate tissue repair during sterile injury, excessive accumulation of extracellular HMGB1 in tissue or circulation contributes importantly to the pathogenesis of many inflammatory or autoimmune diseases, such as sepsis and colitis (Andersson & Tracey, 2011). Notably, genetic deletion of NLRP3 or ASC, two essential components of the NLRP3 inflammasome, blocked LPS-, ATP- or MSU-induced HMGB1 release in cultured macrophages or during endotoxemia (Lamkanfi et al., 2010). Accordingly, the deletion of NLRP3 or ASC confers significant protection against lethal endotoxemia (Mariathasan et al., 2006; Mariathasan et al., 2004). Neutralizing the extracellular HMGB1 using anti-HMGB1 monoclonal antibodies significantly increased the survival during lethal endotoxemia or bacterial sepsis (Lamkanfi et al., 2010; Qin et al., 2006; Wang et al., 1999). These findings establish a critical role of the NLRP3 inflammasome - HMGB1 axis in endotoxemia and sepsis.

We and others previously found that ethyl pyruvate, a metabolite derivative, confers significant protection against experimental sepsis- and endotoxemia-induced lethality, and markedly attenuates the disease severity of experimental colitis (Ulloa et al., 2002; Miyaji et al., 2003; Davé et al., 2009). These studies also show that ethyl pyruvate inhibits LPS-induced HMGB1 release from cultured macrophages and during endotoxemia (Ulloa et al., 2002). However, the underlying mechanism by which ethyl pyruvate inhibits HMGB1 release remains unclear. Early works have shown that EP could work as an anti-inflammatory agent. (Yang et al., 2016) Besides, We and others previously show that inflammasome activation could trigger HMGB1 release in vitro and in vivo (Lu et al., 2012; Lamkanfi et al., 2010). Considering pathogenic known role of the NLRP3 inflammasome in endotoxic shock (Mariathasan et al., 2006; Mariathasan et al., 2004), together with EP’s anti-inflammatory effect, here we postulate that ethyl pyruvate might inhibit HMGB1 release and thus play a protective role in sepsis- and endotoxemia-induced lethality by inhibiting the NLRP3 inflammasome activation.

## Methods

### Reagents

Ultra-pure LPS and the NLRP3 inflammasome agonists ATP, Nigericin,Nano-SiO_2_, Alum Crystals were obtained from Invivogen (San Diego, California). Ethyl pyruvate (EP) was purchased from ThermoFisher Scientific(Shanghai, China). Anti-mouse IL-1β (AF-401-NA) was from R&D and anti-mouse caspase-1 (sc-514) was from Santa Cruz. Mouse HMGB1 mAb IgG2b 2G7 (noncommercial antibody), originally from Critical Therapeutic Inc. (Boston, MA, USA) is available upon request. Acridine orange was purchased from SigmaAldrich (St. Louis, MO). MitoSox™ was purchased from ThermoFisher Scientific (Shanghai, China), and JC-1 from Beyotime (C2006). Mitochondrial isolation and purification kits were from QIAGEN (Hilden, Germany). Murine macrophage colony-stimulating factor (GM-CSF) and human macrophage colony-stimulating factor (M-CSF) were purchased from PEPROTECH (New Jersey, USA).

### Cell preparation and stimulation

#### Peritoneal mouse macrophages

Peritoneal mouse macrophages C57BL/6 mice were isolated in 10% sucrose solution following bilateral injection of 1 ml of thioglycolate bilaterally 3 days prior. Cells were cultured in RPMI medium 1640 supplemented with 10% FBS, 100 U/mL penicillin, and 100 μg/mL streptomycin. One million peritoneal macrophages, plated in 12-well plates, were primed with ultra-pure LPS (1 μg/ml) for 3 h in the presence or the absence of EP (1 and 5 mM), and then stimulated with ATP(5 mM, 30 min), Nigericin (10 μM,1 h), Nano-SiO2 (10μg/ml, 6h) or Alum Crystals (20μg/ml, 6h). For Salmonella infection, wild-type S. typhimurium was grown overnight in Luria–Bertani (LB) broth, then reinoculated at a dilution of 1:100 and grown to mid-exponential phase (3 h) to induce expression of the Salmonella pathogenicity island 1 type III secretion system. To minimize the involvement of NLRP3 inflammasome activation during Salmonella infection, unprimed macrophages were infected with wild-type S. typhimurium (m.o.i. is from 5 to 100). The supernatant samples were collected 1 h after infection. To study AIM2 inflammasome activation, macrophages were transfected with random DNA using Lipofectamine 2000 at a concentration of 1 mg DNA plus 3.5 ml lipofectamine 2000 per ml. The supernatant samples were collected 6 h after transfection.

#### Human macrophages

Primary blood mononuclear cells were isolated by density-gradient centrifugation. Then cells were collected and cultured in RPMI 1640 medium with 10% heat-inactivated human serum. After 2 h incubation at 37 °C, Adherent cells were detached with 10 mM EDTA, and then re-suspended (10^6^ cells/ml) in medium supplemented with human macrophage colony-stimulating factor (20 ng/ml), and cultured for 7 d.

#### Human acute monocytic leukemia cell lines (THP-1)

Human acute monocytic leukemia cell lines (THP-1)were cultured in RPMI medium 1640 supplemented with 10% FBS, 100 U/mL penicillin, and 100 μg/mL streptomycin. When cells were 70% confluence, treatment was carried out in RPMI medium 1640.

#### Cytotoxicity assay

Cell supernatants were analyzed using a lactate dehydrogenase (LDH) cytotoxicity assay kit purchased from TAKARA (California, USA) per manufacture recommendations.

#### Elisa

Levels of IL-1β, and TNF-α in the culture medium were determined using quantitative ELISA kits (R & D Systems, Minneapolis, MN, USA) and HMGB1 (IBL International) according to the manufacturer’s instructions.

#### Western-blot analysis

Supernatant and cell lysates were analyzed using western blot for caspase-1 p10, IL-1β, and HMGB1 release. Proteins from cell-free supernatants were extracted by methanol/chloroform precipitation as previously described. Briefly, cell culture supernatants were precipitated by the addition of an equal volume of methanol and 0.25 volumes of chloroform, then were vortexed and centrifuged for 10 min at 20,000 g. The upper phase was discarded and 500 μl methanol was added to the interphase. This mixture was centrifuged for 10 min at 20,000 g and the protein pellet was dried at 55 °C, re-suspended in Laemmli buffer and boiled for 5 min at 99 °C. Cell extracts were prepared as described previously (Wang et al., 2004). Samples were separated by 4–20% SDS-PAGE or 4–20% native-PAGE and were transferred onto PVDF membranes. The relative band intensity was quantified by using the NIH image 1.59 software to determine HMGB1 levels regarding standard curves generated with purified HMGB1 as described previously.

#### Intracellular K+ concentration detection

Cells were washed twice with 0.9% Nacl and collected after appropriate treatments. Then, 1 × 106 cells from each group were resuspended in 300ul ddH2O. Then the cells were subjected to there Freeze/thaw cycles. Supernatant were collected and used for potassium measurement. Potassium in these samples are assessed by a automatic biochemical analyser (ABBOTT ARCHITECT C16000).

#### Flow cytometry analysis

Mitochondrial ROS production, lysosomal rupture and mitochondrial membrane potential were assessed by flow cytometry. Briefly, to measure Mitochondrial ROS production, cells were primed with ultra-pure LPS (1 μg/ml) for 3 h in the presence of EP (5 mM), followed by treatment with ATP (5 mM,30 min) or Nigericin (10 μM,1 h). Staining treated cells with MitoSOX™ (2.5 μM) for 15 min at 37 °C. For lysosomal rupture measuring, Peritoneal mouse macrophageswere primed with ultra-pure LPS (1 μg/ml) for 3 h in the presence of EP (5 mM), and then stained with Acridine Orange (1μg/ml) for 30 min.After staining, stimulated cells with Alum (20μg/ml) or Nano-SiO_2_(10μg/ml) 6 h. After treatment, cells were collected and quickly transferred on ice for FACS analysis. To determine mitochondrial potential-dependent damage, cells were stained with JC-1 according to the manufacturer’s protocol. Cells were then monitored and analyzed by a flow cytometer (FACS Verse, BD Biosciences).

#### Mitochondrial DNA release assay

1 × 10^7^ peritoneal macrophages were homogenized with a TB syringe, and then were subjected to centrifugation at 2000 *g* for 10 min at 4 °C. Protein concentration and volume of the supernatant were normalized, followed by centrifugation at 6000 *g* for 10 min at 4 °C to produce a supernatant corresponding to the cytosolic fraction. DNA was isolated from 200 μl of the cytosolic fraction using a QIAamp DNA Minikit purchased from QIAGEN (Hilden, Germany). The levels of mtDNA encoding cytochrome *c* oxidase 1 were measured by quantitative real-time PCR with same volume of the DNA solution. The following primers were used: mouse cytochrome *c* oxidaseI forward, 5′-GCCCCAGATATAGCATTCCC-3′, and reverse, 5′-GTTCATCCTGTTCCTGCTCC-3′.

#### Electron microscopy (EM)

Electron micrographs of mitochondria in LPS-primed THP-1 cells were taken after incubation with ATP or nigericin for 15 min in the presence or the absence of ethyl pyruvate (5 mM) using HITACHITransmissionElectronMicroscopeH7700 (HITACHI, Japan). Objects were magnified 15 thousand times and macrographs of mitochondria were collected by gatan ORIUS CCD CAMERA.

#### Statistical analysis

Data in the figures and text are expressed as mean ± SEM of at least three independent experiments (*n* = 3–6). Significance of difference between groups was determined by two-tailed Students *t-*test*.* A *p* value < 0.05 was considered statistically significant.

## Results

### Ethyl pyruvate inhibits NLRP3 agonists-induced inflammasome activation in mouse macrophages

**Fig. 1:**
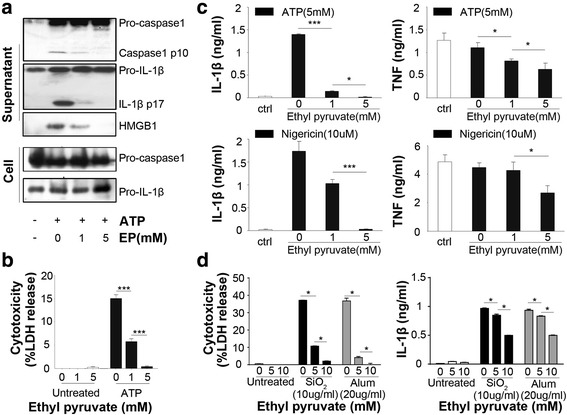
Ethyl pyruvate inhibits NLRP3 agonists-induced inflammasome activation in mouse macrophages. **a** Peritoneal mouse macrophages were primed with ultra-pure LPS (1 μg/ml) for 3 h. in the presence or the absence of EP (1 or 5 mM), and then stimulated with ATP (5 mM) for 30 min. The pro-caspase1, cleavage of caspase-1 and Pro-IL-1β, IL-1β and the release of HMGB1 in supernatants and expression of pro-caspase1, pro-IL-1β in cell were assessed by Western-blot. **b** Peritoneal mouse macrophages were primed with ultra-pure LPS (1 μg/ml) for 3 h. in the presence or the absence of EP (1 or 5 mM), and then stimulated with ATP (5 mM) for 30 min. Cytotoxicity was assessed by lactate dehydrogenase (LDH) assay. **c** Peritoneal mouse macrophages were primed with ultra-pure LPS (1 μg/ml) for 3 h. in the presence or the absence of EP (1 or 5 mM), and then stimulated with ATP (5 mM) or nigericin (10 μM) for 30 min. Levels of IL-1β and TNF-α in the culture medium were determined by ELISA. **d** Peritoneal mouse macrophages were primed with ultra-pure LPS (1 μg/ml) for 3 h. with or without EP (5 or 10 mM), and then stimulated with alum (20 μg/ml) or silica (10 μg/ml) 6h. Cytotoxicity was analyzed by LDH assay and IL-1β level was determined by ELISA. Results are means ± SEM (*n* = 3). *p < 0.05; **p < 0.01; ***p < 0.001

To determine whether ethyl pyruvate (EP) inhibits the NLRP3 inflammasome activation, LPS-primed mouse peritoneal macrophages were stimulated with ATP in the presence or the absence of different concentrations of EP. EP exposure dose-dependently inhibited ATP-induced activation of caspase-1, cleavage of pro-IL-1β and HMGB1 release (Fig. 1a). Addition of EP failed to inhibit the expression of pro-IL-1β in the cell lysate (Fig. 1a), indicating that the inhibition of IL-1β production by EP is due to the suppression of inflammasome activation, rather than LPS-induced priming. Further, we observed that EP dose-dependently inhibited ATP-induced pyroptosis in LPS-primed mouse peritoneal macrophages, as showed by LDH assay (Fig. 1b).

To test whether EP inhibits the NLRP3 inflammasome activation induced by NLRP3 agonists other than ATP, LPS-primed mouse peritoneal macrophages were stimulated with nigericin, a known potassium ionophore in the presence of absence of different concentrations of EP. Notably, EP exposure significantly inhibited IL-1β expression at the concentration of 5 mM. EP slightly inhibited TNF at the concentration of 5 mM, suggesting that EP more specifically inhibits NLRP3-dependent cytokine release (Fig. 1c). Furthermore, EP dose-dependently inhibits IL-1β production and pyroptosis in LPS-primed mouse peritoneal macrophages induced by silica and Alum crystal (Fig. 1d). Intriguingly, EP showed weaker inhibitory effect in crystals-induced NLRP3 inflammasome activation than that induced by ATP or NIG. Taken together, these results indicate that EP inhibits the NLRP3 inflammasome activation in mouse macrophages.

### Ethyl pyruvate specifically inhibits the NLRP3 inflammasome activation

**Fig. 2:**
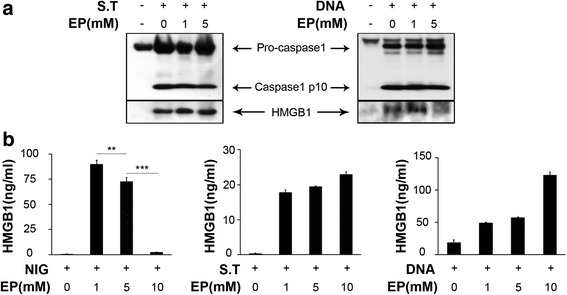
Ethyl pyruvate specifically inhibits the NLRP3 inflammasome activation. For Salmonella infection, unprimed macrophages were infected with wild-type S. typhimurium and the supernatant samples were collected 1 h after infection. For the AIM2 inflammasome activation, macrophages were transfected with DNA using Lipofectamine 2000 and the supernatant samples were collected 6 h after transfection. **a** Pro-caspase1, caspase1 and HMGB1 release were assessed by Western-blot. **b** Supernatant levels of HMGB1were determined by ELISA. Results are means ± SEM (n = 3). *p < 0.05; **p < 0.01; ***p < 0.001

To address the specificity of EP in inhibiting the NLRP3 inflammasome activation, we next investigated whether EP inhibits the activation of AIM2 or NLRC4 inflammasome. Mouse peritoneal macrophages were either primed with LPS and then stimulated with nigericin, or directly stimulated with salmonella typhimurium (ST), a known NLRC4 inflammasome agonist (Mariathasan et al., 2004), or transfected with poly(dA-dT). poly(dA-dT) (hereafter termed poly (dA: dT)), a known AIM2 inflammasome agonist (Hornung et al., 2009; Fernandes-Alnemri et al., 2009). Consistently, EP exposure dose-dependently inhibited HMGB1 release in nigericin-treated macrophages. However, the addition of EP failed to inhibit caspase-1 activation and HMGB1 release induced by salmonella typhimurium infection or poly(dA:dT) transfection (Fig. 2a-b). These results indicate that EP specifically inhibits the NLRP3 inflammasome activation.

### Inhibition of NLRP3 activation by EP is independent of potassium efflux

**Fig. 3:**
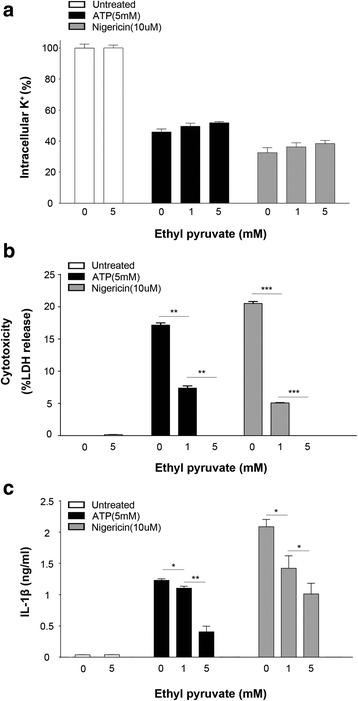
Inhibition of NLRP3 activation by EP is independent of potassium efflux. Peritoneal mouse macrophages were primed with ultra-pure LPS (1 μg/ml) for 3 h. with or without the presence of EP (1 or 5 mM), and then stimulated with ATP (5 mM) or nigericin (10 μM) for 30 min. **a** Intracellular K+ concentration was measured. **b** Cytotoxicity was assessed by lactate dehydrogenase (LDH) cytotoxicity assay. **c** Levels of IL-1β in the culture medium was determined by ELISA. Results are means ± SEM (n = 3). *p < 0.05; **p < 0.01; ***p < 0.001

A role of potassium (K^+^) efflux in NLRP3 activation has been proposed since several NLRP3 activators including ATP, NIG, MSU and particulate matters like silica and Alum crystal trigger the efflux of K^+^ and preventing K^+^ efflux blocks inflammasome activation induced by NLRP3 agonists (Muñoz-Planillo et al., 2013; He et al., 2016). Therefore, we next determined whether EP inhibits the NLRP3 inflammasome activation through regulating the K^+^ efflux. To test this possibility, LPS-primed mouse peritoneal macrophages were stimulated with ATP or nigericin in the presence or the absence of different concentrations of EP. EP exposure dose-dependently inhibited ATP- or NIG-induced cytotoxicity (%) (Fig. 3b) and IL-1β release (Fig. 3c) whereas failed to affect K^+^ efflux (Fig. 3a). Thus, Inhibition of NLRP3 activation by EP is independent of potassium efflux.

### Inhibition of NLRP3 activation by EP is independent of lysosomal rupture

**Fig. 4:**
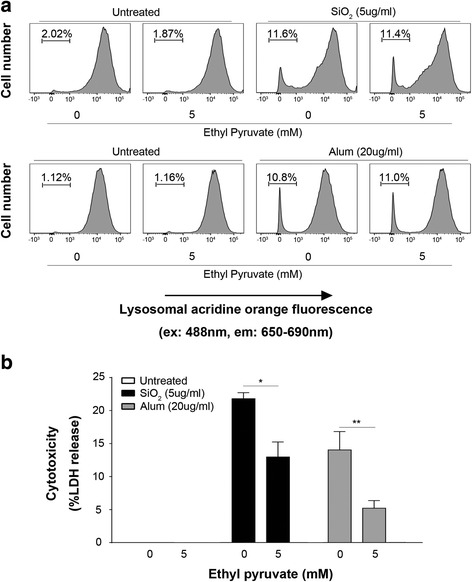
Inhibition of NLRP3 activation by EP is independent of lysosomal rupture. Peritoneal mouse macrophages were primed with ultra-pure LPS (1 μg/ml) for 3 h. in the presence or the absence of 5 mM EP, and then stained with acridine orange (1 μg/ml) for 30 min at 37 °C. Cells were then stimulated by alum (20 μg/ml) or silica (10 μg/ml) 6h. **a** After stimulation, cells were analyzed by flow cytometry for lysosomal acridine orange fluorescence. **b** Cytotoxicity was assessed using LDH assay. Results are means ± SEM (n = 3). *p < 0.05; **p < 0.01; ***p < 0.001

It has been proposed that lysosomal rupture mediates the NLRP3 inflammasome activation induced by particulate matters, such as silica and Alum crystal (Hornung et al., 2008; Halle et al., 2008). Since EP inhibits the NLRP3 inflammasome activation induced by particulate matters including silica and alum crystal, we investigated whether EP inhibits the NLRP3 inflammasome activation through regulating the lysosomal rupture. To test this possibility, LPS-primed mouse peritoneal macrophages were stimulated with silica or Alum crystal in the presence or the absence of different concentrations of EP, and lysosomal rupture was assessed by flow cytometry. Surprisingly, EP exposure failed to prevent, but instead promoted silica- or Alum-induced lysosomal rupture (Fig. 4a), although EP dose-dependently inhibited silica- or Alum-induced cytotoxicity (Fig. 4b). Therefore, inhibition of NLRP3 activation by EP is independent of lysosomal rupture.

### Ethyl pyruvate inhibits NLRP3 agonists-induced mitochondrial damage

**Fig. 5:**
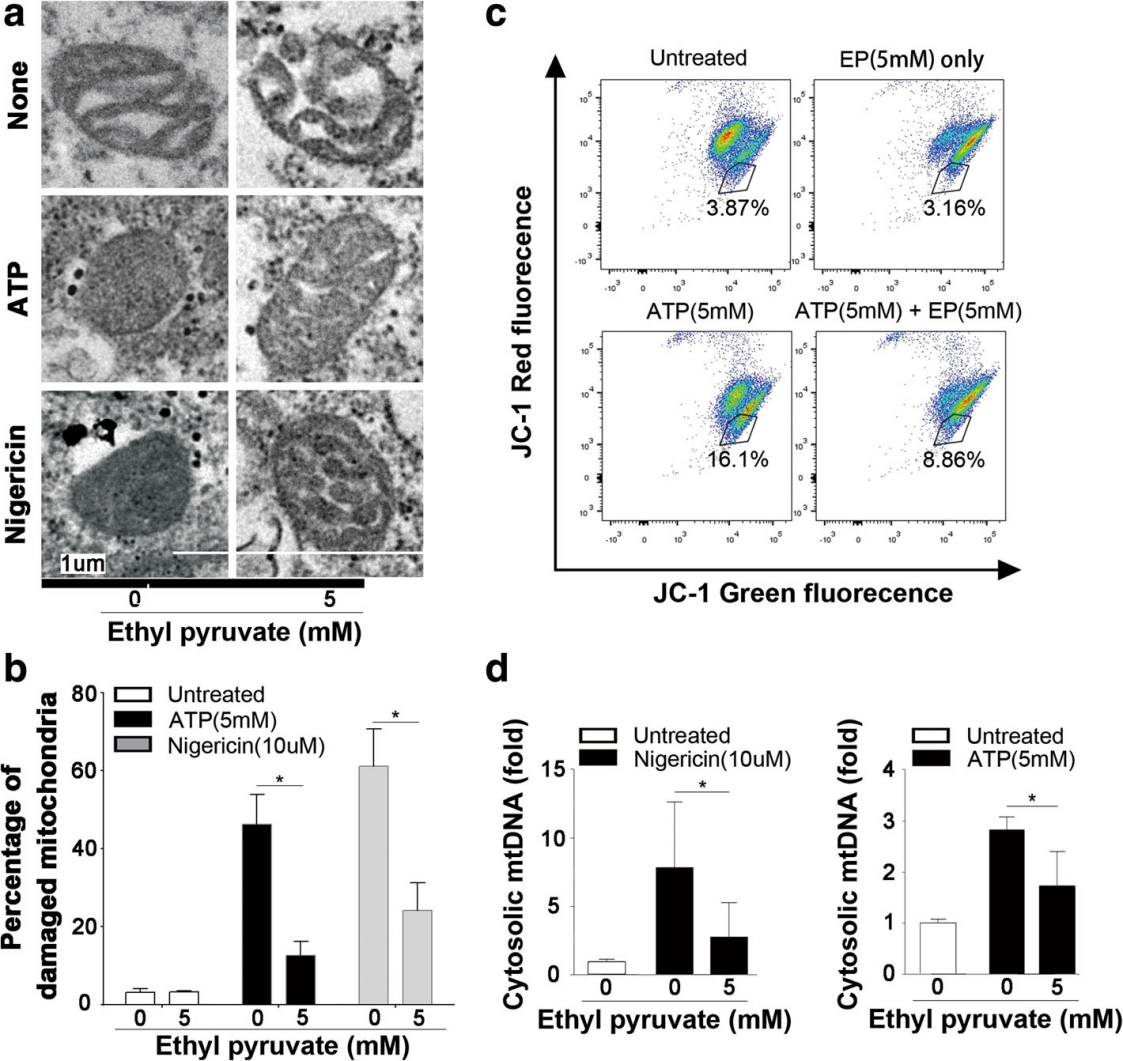
Ethyl pyruvate inhibits NLRP3 agonists-induced mitochondrial damage. **a** Electron micrographs of mitochondria in LPS-primed THP-1 cells after incubation with ATP (5 mM) or nigericin (10 μM) for 15 min in the presence or the absence of ethyl pyruvate (5 mM). Shown in panel A are representative images of normal, partially damaged, or heavily damaged mitochondria. Scale bars, 1 um. **b** Quantification of damaged mitochondria in (**a**). Results are means ± SEM (*n* = 6). **c** Mouse macrophages were primed with LPS or LPS + EP, followed by treatment with ATP (5 mM, 20 min). Cells were stained with JC-1 and analyzed by a flow cytometer. Cells in the gate represent mitochondrial depolarization or mitochondrial damage. **d** mtDNA release from LPS-primed mouse peritoneal macrophages after stimulation of ATP (5 mM) or nigericin (10 μM) for 30 min in the presence or the absence of ethyl pyruvate (5 mM). Results are means ± SEM (n = 3). *p < 0.05; **p < 0.01; ***p < 0.001

In addition to K^+^ efflux and lysosomal rupture, accumulated evidence show that mitochondrial damage is critical for the NLRP3 inflammasome activation (Zhou et al., 2011; Nakahira et al., 2011; Shimada et al., 2012; Zhong et al., 2016). To test whether ethyl pyruvate prevents NLRP3 agonists-triggered mitochondrial damage, we directly assessed mitochondrial integrity in human acute monocytic leukemia cell lines (THP-1) using electron microscopy (EM). Whereas nigericin, and to a lesser extent, ATP induced accumulation of many highly damaged, electron-dense mitochondria, this effect was strongly inhibited by ethyl pyruvate in the concentration of 5 mM (Fig. 5a-b). To futher confirm the finding that EP significantly relieves mitochondrial damage, we employed JC-1, a dye used to detect the mitochondrial membrane potentialmeasure and assess mitochondrial integrity. The JC-1 red fluorecence decreased and JC-1 green fluorecence increased when mitochondrial damage occurs. EP could reduce the proportion of mitochondrial damaged cells (Fig. 5c), in accordance with the results by electron microscopy.

Early studies indicate that mitochondrial DNA (mtDNA) release into the cytoplasm is highly linked to the NLRP3 inflammasome activation. Oxidized mtDNA was reported to function as a direct NLRP3 ligand that induces the assembly and activation of the NLRP3 inflammasome (Shimada et al., 2012). Accordingly, we reasoned that EP might prevent mtDNA release into the cytoplasm. To test this hypothesis, mouse macrophages were stimulated with ATP or nigericin in the presence or the absence of EP. The cytoplasmic fraction was analyzed for mtDNA levels using qPCR with primers for cytochrome C oxidase-1 gene. Indeed, EP inhibits ATP- or nigericin-induced mtDNA release into the cytoplasm (Fig. 5d). These results suggest that the mechanisms by which EP inhibits the NLRP3 inflammasome activation, are at least in part, through the inhibition of mitochondrial damage.

### Ethyl pyruvate does not inhibit NLRP3 agonists-induced mtROS production

**Fig. 6:**
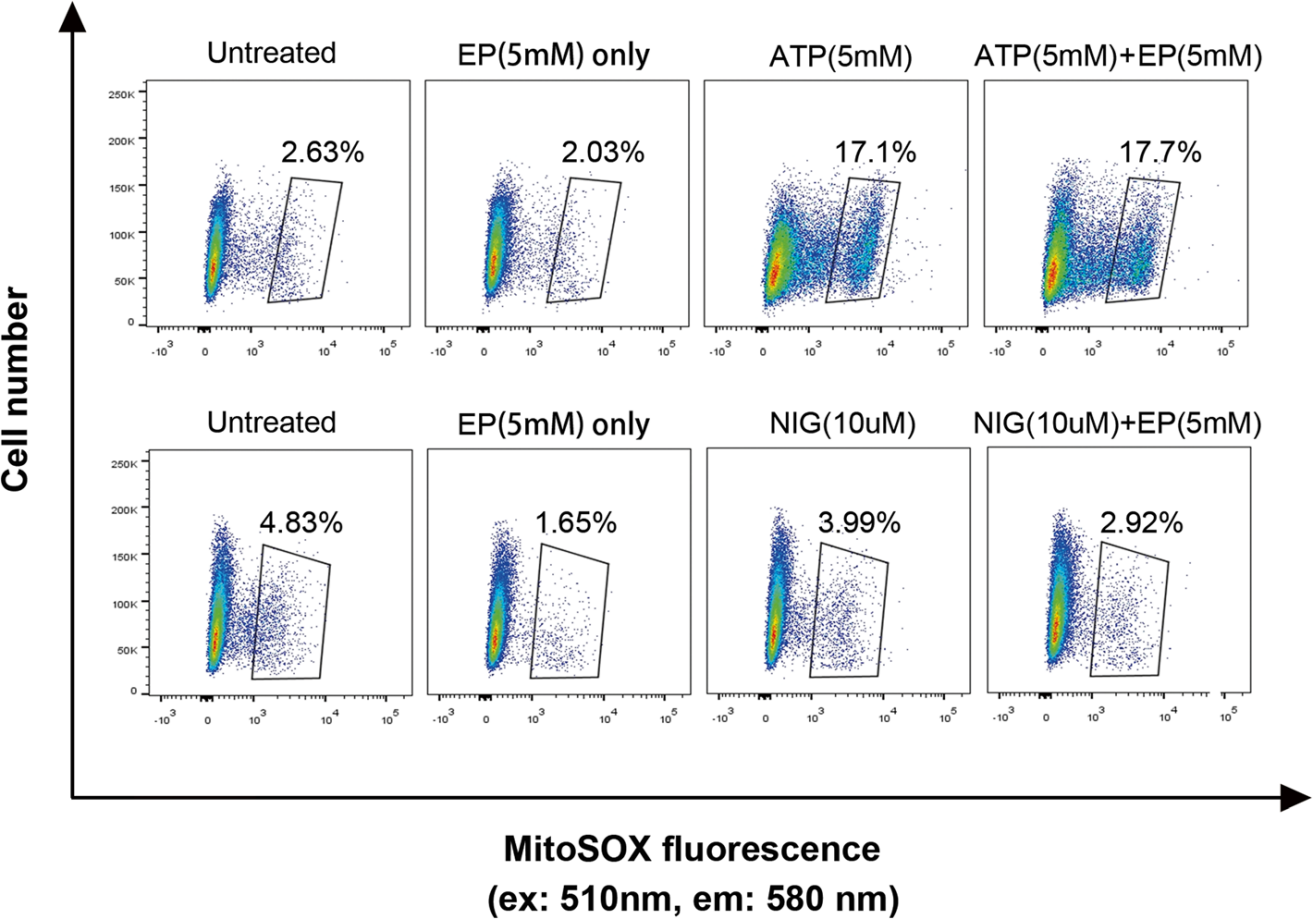
Ethyl pyruvate does not inhibit NLRP3 agonists-induced mtROS production. MtROS levels were determined by MitoSOX staining of LPS-primed human acute monocytic leukemia cell lines (THP-1) after stimulation with ATP (5 mM) or nigericin (10 μM) in the presence or the absence of 5 mM EP

Early studies indicate that mitochondrial reactive oxygen species (mtROS) production contributes to mitochondrial damage and the NLRP3 inflammasome activation (Zhou et al., 2011). Accordingly, we next tested whether EP inhibits NLRP3 agonists-induced mtROS production in mouse macrophages. A robust mtROS generation was detected when treated with ATP while little mtROS was induced when treated with nigericin. Notably, EP did not inhibit mtROS production in these cells as measured by flow cytometry (Fig. 6). Together with the finding that EP inhibits NLRP3 agonists-induced mitochondrial damage, these observations suggest that the mechanism through which EP prevents mitochondrial damage and the NLRP3 inflammasome activation is independent of mtROS production.

### Ethyl pyruvate inhibits NLRP3 agonists-induced inflammasome activation in human macrophages

**Fig. 7:**
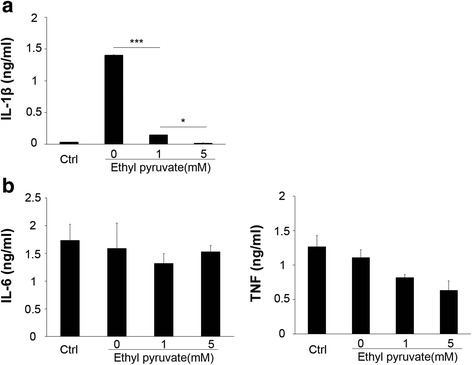
Ethyl pyruvate inhibits NLRP3 agonists-induced inflammasome activation in human macrophages. Human macrophages were primed with ultra-pure LPS (1 μg/ml) for 3 h. in the presence or the absence of EP (1 or 5 mM), and then stimulated with ATP (5 mM) for 30 min. **a** IL-1β level and (**b**) IL-6, TNF production were determined by ELISA. Results are means ± SEM (n = 3). *p < 0.05; **p < 0.01; ***p < 0.001

We finally examined whether EP inhibits the NLRP3 inflammasome activation in human macrophages. ATP stimulation of LPS-primed human macrophages rapidly induced IL-1β secretion, which was dose-dependently inhibited by EP (Fig. 7a). In contrast, addition of EP at 1 and 5 mM failed to attenuate LPS-induced production of non-NLRP3 inflammasome dependent cytokines, such as IL-6 or TNF production (Fig. 7b). This suggests that EP inhibits the NLRP3 inflammation activation in both murine and human macrophages.

## Discussion

Ethyl pyruvate is a small molecule and a food additive that exerts anti-inflammatory effect. We and others previously show that treatment with ethyl pyruvate is able to ameliorate systemic inflammation and prevents multiple organ dysfunctions in a number of disorders, including endotoxemia, bacterial sepsis, acute pancreatitis, alcoholic liver injury, acute respiratory distress syndrome, acute viral myocarditis and acute kidney injury. However, the underlying mechanisms by which ethyl pyruvate attenuates systemic inflammation remain elusive. In this study, we first show that ethyl pyruvate inhibits NLRP3 agonists-induced inflammasome activation through preventing mitochondrial damage. Interestingly, ethyl pyruvate neither inhibits the activation of AIM2 or NLRC4 inflammasome nor affects the inflammatory responses that are not related to the inflammasome activity.

The NLRP3 inflammasome is an intracellular protein complex comprised of NLRP3, ASC and caspase-1. Upon stimulation by a variety of endogenous or exogenous danger signals, these inflammasome components rapidly assemble into the active NLRP3 inflammasome, cumulating in the maturation of IL-1β and IL-18, as well as pyroptosis, a lytic form of programmed cell death (Strowig et al., 2012). The NLRP3 inflammasome importantly orchestrates host innate immune responses to infections or sterile injuries. Deregulated NLRP3 inflammasome activity, however, contributes to the pathogenesis of a number of human diseases or life-threatening conditions, such as endotoxemia, sepsis, alcoholic liver injury, colitis and acute kidney injury. Together with findings in current study, these observations could explain why ethyl pyruvate is able to confer protection in various diseases or life-threatening conditions. It is noteworthy that ethyl pyruvate is well-known to inhibit HMGB1 release from active immune cells or in inflammatory diseases (Ulloa et al., 2002; Miyaji et al., 2003; Davé et al., 2009). HMGB1 is a proinflammatory mediator that contributes to the pathogenesis of many disorders, including sepsis and colitis. We and others recently found that the release of HMGB1 is an important downstream event of inflammasome activation (Nakahira et al., 2011; Groß et al., 2016). Neutralizing extracellular HMGB1 with monoclonal antibodies confers considerate protection against Caspase-1/Caspase-11-mediated lethality during endotoxemia (Lamkanfi et al., 2010). Consistent with these findings, we observed in this study that ethyl pyruvate dose-dependently inhibits the NLRP3 agonists-induced HMGB1 release.

Though the NLRP3 inflammasome has been extensively studied due to its important roles in immune responses and diseases, how various endogenous or exogenous danger signals activate the NLRP3 inflammasome still remains largely unknown. In recent years, several cellular events have been proposed to be the triggers for the NLRP3 inflammasome activation. These include potassium efflux, lysosomal rupture, mitochondrial reactive oxygen species (ROS) production and mitochondrial damage. Potassium efflux could be induced by most NLRP3 stimuli, and therefore has been proposed to be the common pathway for the NLRP3 inflammasome activation. However, ethyl pyruvate has no detectable effect on the ATP- or nigericin-induced potassium efflux at the concentration, when it could completely abolish ATP- or nigericin-induced inflammasome activation. These observations suggest that ethyl pyruvate inhibits the NLRP3 inflammasome activation through affecting the downstream signal of potassium efflux. Disruption of the lysosomal membrane, caused by phagocytosis of particulate matter such as silica or Alum crystals, could also activate the NLRP3 inflammasome. Though ethyl pyruvate could effectively inhibit silica- or Alum-induced inflammasome activation, it barely affects the phagosomal rupture-induced by silica- or Alum. Further, we also noticed that ethyl pyruvate fails to inhibit ATP-induced mitochondrial ROS production. A number of studies demonstrate that mitochondrial ROS is critical for the NLRP3 inflammasome activation (Zhou et al., 2011; Nakahira et al., 2011; Groß et al., 2016). However, some other studies indicate that mitochondrial ROS is dispensable for the activation of NLRP3 inflammasome (Muñoz-Planillo et al., 2013; Bauernfeind et al., 2011; Won et al., 2013). One explanation for the discrepancy is that mitochondrial ROS activate the NLRP3 inflammasome in the context-dependent manner, which suggests that the downstream events of mitochondrial ROS might be the actual trigger for the NLRP3 inflammasome activation.

Excessive mitochondrial ROS could lead to mitochondrial damage. Accumulated evidences show an essential role of mitochondrial damage in the NLRP3 inflammasome activation (Zhong et al., 2016; Wang et al., 2014; Shimada et al., 2012; Iyer et al., 2013). Iyer et al. show that the cytosolic oxidized mitochondrial DNA released by damaged mitochondria serve as NLRP3 ligands and could induce the NLRP3 inflammasome activation (Shimada et al., 2012). Another study demonstrates that the cardiolipins exposed on the surface of damaged mitochondria can directly bind NLRP3 and play critical roles in the activation of NLRP3 inflammasome (Iyer et al., 2013). As show in current study, ethyl pyruvate prevents mitochondrial damage and inhibits the release of mitochondrial DNA into the cytosol in NLRP3 agonist-stimulated macrophages. Though whether cytosolic oxidized mitochondrial DNA is a bona fide NLRP3 ligand requires further investigation, our findings suggest that ethyl pyruvate inhibits the NLRP3 inflammasome activation, at least in part, through preventing mitochondrial damage.

## Conclusion

Together, our findings establish ethyl pyruvate as a novel NLRP3 inhibitor and unravel the mechanisms by which this metabolite derivative exerts anti-inflammatory effect in various types of diseases or illnesses, such as sepsis, alcoholic liver injury, and acute kidney injury. Mechanistically, ethyl pyruvate inhibits the NLRP3 inflammasome activation, at least in part, by reducing mitochondrial damage.

## References

[CR1] Andersson U, Tracey KJ (2011). HMGB1 is a therapeutic target for sterile inflammation and infection. Annu Rev Immunol.

[CR2] Bauernfeind F (2011). Cutting edge: reactive oxygen species inhibitors block priming, but not activation, of the NLRP3 inflammasome. J Immunol.

[CR3] Davé SH (2009). Ethyl pyruvate decreases HMGB1 release and ameliorates murine colitis. J Leukoc Biol.

[CR4] Fernandes-Alnemri T, Yu JW, Datta P, Wu J, Alnemri ES (2009). AIM2 activates the inflammasome and cell death in response to cytoplasmic DNA. Nature.

[CR5] Groß CJ (2016). K+ efflux-independent NLRP3 Inflammasome activation by small molecules targeting mitochondria. Immunity.

[CR6] Halle A (2008). The NALP3 inflammasome is involved in the innate immune response to amyloid-beta. Nat Immunol.

[CR7] He Y, Zeng MY, Yang D, Motro B, Núñez G (2016). NEK7 is an essential mediator of NLRP3 activation downstream of potassium efflux. Nature.

[CR8] Hornung V (2008). Silica crystals and aluminum salts activate the NALP3 inflammasome through phagosomal destabilization. Nat Immunol.

[CR9] Hornung V, et al. AIM2 recognizes cytosolic dsDNA and forms caspase-1-activating inflammasome with ASC. Nature. 2009;458(7237):514–8. 10.1038/nature07725 PMC2726264 19158675

[CR10] Iyer SS (2013). Mitochondrial cardiolipin is required for Nlrp3 inflammasome activation. Immunity.

[CR11] Lamkanfi M (2010). Inflammasome-dependent release of the alarmin HMGB1 in endotoxemia. J Immunol.

[CR12] Lu B (2012). Novel role of PKR in inflammasome activation and HMGB1 release. Nature.

[CR13] Mariathasan S (2004). Differential activation of the inflammasome by caspase-1 adaptors ASC and Ipaf. Nature.

[CR14] Mariathasan S (2006). Cryopyrin activates the inflammasome in response to toxins and ATP. Nature.

[CR15] Miyaji T (2003). Ethyl pyruvate decreases sepsis-induced acute renal failure and multiple organ damage in aged mice. Kidney Int.

[CR16] Muñoz-Planillo R (2013). K^+^ efflux is the common trigger of NLRP3 inflammasome activation by bacterial toxins and particulate matter. Immunity.

[CR17] Nakahira K (2011). Autophagy proteins regulate innate immune responses by inhibiting the release of mitochondrial DNA mediated by the NALP3 inflammasome. Nat Immunol.

[CR18] Qin S (2006). Role of HMGB1 in apoptosis-mediated sepsis lethality. J Exp Med.

[CR19] Rittirsch D (2008). Functional roles for C5a receptors in sepsis. Nat Med.

[CR20] Schroder K, Tschopp J (2010). The inflammasomes. Cell.

[CR21] Shimada K (2012). Oxidized mitochondrial DNA activates the NLRP3 inflammasome during apoptosis. Immunity.

[CR22] Strowig T, Henao-Mejia J, Elinav E, Flavell R (2012). Inflammasomes in health and disease. Nature.

[CR23] Ulloa L (2002). Ethyl pyruvate prevents lethality in mice with established lethal sepsis and systemic inflammation. Proc Natl Acad Sci U S A.

[CR24] Wang H (1999). HMG-1 as a late mediator of endotoxin lethality in mice. Science.

[CR25] Wang H (2004). Cholinergic agonists inhibit HMGB1 release and improve survival in experimental sepsis. Nat Med.

[CR26] Wang X (2014). RNA viruses promote activation of the NLRP3 inflammasome through a RIP1-RIP3-DRP1 signaling pathway. Nat Immunol.

[CR27] Won J-H (2013). Rotenone-induced impairment of mitochondrial electron transport chain confers a selective priming signal for NLRP3 Inflammasome activation. J Biol Chem.

[CR28] Yang R, Zhu S, Tonnessen TI (2016). Ethyl pyruvate is a novel anti-inflammatory agent to treat multiple inflammatory organ injuries. J Inflamm.

[CR29] Zhong Z (2016). NF-κB restricts Inflammasome activation via elimination of damaged mitochondria. Cell.

[CR30] Zhou R, Yazdi AS, Menu P, Tschopp J (2011). A role for mitochondria in NLRP3 inflammasome activation. Nature.

